# An Essay on the Nature and the Consequences of Anomalies of Refraction

**Published:** 1899-06

**Authors:** 


An Essay on the Nature and the Consequences of Anomalies of Refraction. By F. C. Donders, M. D., Late Professor of Physiology and Ophthalmology in the University of Utrecht. (Translated under the supervision of the Kirschbaum School of Languages and Bureau of Translation of Philadelphia.) Revised and edited by Charles A. Oliver, A. M., M. D. (Univ. Pa.), one of the Attending Surgeons to the Wills' Eye Hospital; one of the Ophthalmic Surgeons to the Philadelphia Hospital, etc. With portrait and other illustrations. Duodecimo, pp. eighty-two. Philadelphia: P. Blakiston's Son & Co. 1899.

Donders's classic on the anomalies of refraction and accommodation, translated into English and published in 1864, has long been out of print. The present volume will in a small degree take the place of the larger work, as it is substantially a summary of it by Donders, himself. Dr. Oliver has done the profession a great favor in thus presenting what may properly be termed the aphorisms of Donders.

The volume is a small one of 82 pages, tastefully printed and bound, and contains an excellent portrait of the author. Even at the present time, oculists will profit by a study of Donders' findings as here set forth. A. A. H.

